# Evaluation of Cardiovascular Concerns of Intravenous Lacosamide Therapy in Epilepsy Patients

**DOI:** 10.3389/fneur.2022.891368

**Published:** 2022-07-04

**Authors:** Yan-Ting Lu, Chih-Hsiang Lin, Chen-Jui Ho, Che-Wei Hsu, Meng-Han Tsai

**Affiliations:** ^1^Department of Neurology, Kaohsiung Chang Gung Memorial Hospital, College of Medicine, Chang Gung University, Kaohsiung, Taiwan; ^2^School of Medicine, College of Medicine, Chang Gung University, Taoyuan, Taiwan; ^3^Genomics and Proteomics Core Laboratory, Department of Medical Research, Kaohsiung Chang Gung Memorial Hospital, Kaohsiung, Taiwan

**Keywords:** lacosamide, clinical cardiac safety, sodium channel blocker, antiseizure drugs, arrhythima

## Abstract

**Objective:**

Voltage-gated sodium channels (VGSCs) play an important role in neuronal excitability and epilepsies. In addition to the brain, VGSCs are also abundant enriched in cardiac tissues and are responsible for normal cardiac rhythm. Theoretically, sodium channel blocking antiseizure medications (SCB-ASMs) may have unwanted cardiac side effects. Lacosamide (LCM) is increasingly used in patients with status epilepticus (SE) due to the availability of intravenous formula. The concerns about the proarrhythmic effect are even higher due to the need for rapid administration of LCM. There were limited data on the cardiac safety of intravenous LCM. Hereby, we performed a study to observe the effect of intravenous loading of LCM in patients with seizures in our Neurological Intensive Care Unit (NICU).

**Methods:**

We retrospectively reviewed the patients using parenteral LCM for seizures in NICU. A routine infusion time of 30 min was performed. The electrocardiogram (ECG) and blood pressure were recorded before and after LCM injection.

**Results:**

We retrospectively reviewed the clinical data of 38 patients using LCM for treating seizures. Two patients had cardiac side effects after LCM loading, one (3.0%) with new-onset first-degree AV block and the other (3.0%) with atrial premature complex. For the quantitative changes of ECG parameter analysis, there was no change in QRS complex, corrected QT intervals, and heart rate except that the PR interval was mildly increased. A mild decrease in the diastolic blood pressure and mean arterial pressure were also observed. None of the above-mentioned parameter alterations required clinical intervention.

**Conclusion:**

We evaluated the cardiac safety concern in real-world epilepsy patients requiring intravenous LCM. Near half of this cohort responded to LCM therapy and there was no life-threatening cardiac adverse effect. Intravenous LCM does have some effects on the ECG parameters and blood pressure but without clinical relevance. Despite the theoretical concern of cardiac adverse effects of LCM, the benefit of seizure control outweighed the risk in patients with status epilepticus or seizure clusters, such as hyperthermia, pulmonary edema, cardiac arrhythmias, or cardiovascular collapse.

## Introduction

Voltage-gated sodium channels (VGSC) are important ion channels that are responsible for the generation of action potentials (1). VGSCs are known to play an important role in neuronal excitability and epilepsies; especially mutations in VGSC subunit genes such as *SCN1A, SCN2A, SCN3A*, and *SCN8A* have been found to cause human epilepsies (2). In addition, many current antiseizure medications (ASMs) block the sodium channels through enhancement of either fast or slow inactivation (3, 4). In addition to the nervous system, VGSCs also are enriched in cardiac tissues and are responsible for the normal cardiac rhythm (5). Theoretically, sodium channel blocking ASMs (SCB-ASMs) may theoretically have unwanted cardiac side effects.

Recent studies have shown increasing cardiac adverse effects among the patients receiving lacosamide (LCM). Kim et al. (6) observed that about one-third of patients would experience cardiac side effects after loading of LCM, with first-degree AV block being most common in their study. A review study conducted by Yadav et al. (7) revealed ventricular tachycardia was the most common reported arrhythmia. The US Food and Drug Administration (FDA) also issued a warning on one of the sodium channels blocking ASMs, lamotrigine. The warning is based on *in vitro* testing that suggested lamotrigine exhibits Class IB anti-arrhythmic activity that could slow ventricular conduction (QRS complex) and induce cardiac arrhythmia (8). FDA later further required more studies to evaluate the cardiac risk of other medications across the drug class, including carbamazepine, eslicarbazepine, fosphenytoin, lacosamide, oxcarbazepine, phenytoin, rufinamide, topiramate, and zonisamide (9). However, there are debates on this issue, especially lamotrigine did not change ventricular conduction in human studies except for mild PR prolongation (10–13).

Lacosamide is a new ASM and enhances the slow inactivation of the VGSC (4). It has been increasingly used in patients with status epilepticus (SE) due to the availability of intravenous formula (14). Rapid administration of ASMs to terminate seizure activities are crucial in SE that may be related to better cognitive outcomes and overall survival (15). Conversely, the concerns about the proarrhythmic effect of SCB-ASMs are even higher with the rapid loading of LCM. This was known for another old SCB-ASM, phenytoin (16). There were limited data on the cardiac safety of intravenous LCM (6, 17, 18). A Korean study that focused on the cardiac safety of rapid intravenous LCM suggested a relatively higher rate (32.9%) of cardiac adverse event, especially the new-onset first-degree atrioventricular block (22.4%) (6). Hereby, we performed a study to observe the effect of intravenous loading of LCM in patients with seizures in our Neurological Intensive Care Unit (NICU) and concomitant use with other ASMs, anti-hypertensive drugs, or anti-arrhythmia drugs.

## Materials and Methods

### Study Design and Patients

This was a retrospective study that reviewed the medical records of patients older than 20 years old using parenteral LCM for treatment of acute seizures, seizure clusters, and status epilepticus in the NICU at Kaohsiung Chang Gung Memorial Hospital between January 2018 to April 2021. We excluded patients previously using LCM for seizure control. LCM was given after being diluted in 100 cc of 0.9% sodium chloride and infused over 30 min through the volume metric administration set. A loading dose was given after seizure whenever the treating physician considered it appropriate followed by a maintaining dose of LCM twice per day with an interval of 12 h. The dose of the LCM was determined by the treating physician according to experts' recommendations (19). This study was approved by the Chang Gung Medical Foundation Institutional Review Board (IRB No.: 202100716B0).

The clinical information was recorded using a standardized evaluation form, including the onset symptoms, etiology of seizure, seizure treatment, duration needed for seizure termination, concomitant medications, and comorbidities. We also recorded the biochemistry data before the use of LCM.

### Definitions and Criteria

Seizure clusters was defined as ≥2 seizures in a 6-hour time (20). SE was defined as continuous clinical and/or electrographic seizure activity that lasted for at least 5 min, or recurrent seizure activity with no recovery period (returning to baseline) between seizures (21). The patient's seizure frequency does not fulfill the definition of seizure clusters or SE were categorized as having an acute seizure.

Seizure termination was evaluated as the cessation of clinical ictal phenomena and the absence of electrographic seizure in electroencephalography (EEG). The electrographic seizure was defined as: (1) spikes, polyspikes, sharp waves, and sharp-and-slow-wave complexes with frequency >2.5 Hz; (2) spikes, polyspikes, sharp waves, and sharp-and-slow-wave complexes with frequency < 2.5 Hz but EEG improvement after IV ASMs or typical spatiotemporal evolution (22). The last ASM that resulted in seizure termination was considered the one that stopped the seizures. To evaluate the effectiveness of LCM, we recorded the sequence of LCM used in treating seizures and if it is the last ASM that stopped the seizures.

### Evaluation of Cardiac Safety

A routine baseline electrocardiogram (ECG) was acquired when patients were admitted to NICU. After the injection of LCM was completed, a second ECG was performed in <1 h. ECG parameters including the PR interval, heart rate, the duration of QRS complex, and the corrected QT interval were collected and compared before and after LCM use. Routine blood pressure monitoring was performed every 1 h in NICU. Pre-LCM blood pressure was defined as the closest measured value within 1 h before LCM injection, whereas post- LCM blood pressure was defined as the closest measured value within 1 h after LCM injection. Hypotension was defined as a systolic blood pressure <90 mm Hg or a ≥30% reduction from baseline systolic blood pressure. Bradycardia was defined as a heart rate <50 BPM or a ≥30% reduction in baseline heart rate.

### Statistical Analysis

Statistical analyses were performed using the IBM SPSS Statistics for Windows (version 22; IBM Corp., Armonk, NY, USA). The clinical statistics were presented as percentages for categorical data and median plus interquartile range for skewed continuous variables. Categorical variables were assessed using Fisher exact tests and continuous variables were compared using the Mann-Whitney U-test. To compare the changes in the cardiac safety parameters, Wilcoxon Signed-Rank Test was conducted. *p* values < 0.05 were considered statistically significant.

## Results

During the study period (January 2018 to April 2021), 38 patients who received parental LCM were reviewed. The median age of the studied population was 62.0 years old (interquartile range = 35.5–71.25). Seventeen males and 21 females were included. The number of patients treated for acute seizure, seizure clusters, and status epilepticus was six, seven, and twenty-five, respectively. Sixteen patients were taking ASM for underlying epilepsy control and five patients had underlying heart disease, including four patients with atrial fibrillation and one with first-degree AV block. The demographic data of the studied population before the initiation of LCM was presented in Table 1. After the seizure onset, 26 patients received benzodiazepine use, including 24 with lorazepam, one with diazepam, and one with midazolam. During the treatment of seizure, 35 patients had already received other ASMs when LCM was given, levetiracetam was used in 30 patients, valproic acid was used in 25 patients, perampanel was used in six patients, phenytoin and topiramate were used in three patients, lamotrigine was used in two patients, and one patient for each of carbamazepine, phenobarbital, pregabalin, and zonisamide. Other concomitant medications included antiarrhythmic drugs used in seven patients and anti-hypertension drugs used in 10 patients. The detailed concomitant medications used was listed in Table 2. Ten patients were having renal impairment with eight having moderate impairment (eGFR: 30–59.9 ml/min/1.73m^2^), one severe impairment (eGFR: 15–29 ml/min/1.73m^2^), and one end-stage renal disease (eGFR: <15 ml/min/1.73m^2^) with hemodialysis. In our study, only one patient had an abnormal liver function test with two times alanine aminotransferase increment (alanine aminotransferase = 103 U/L) before LCM use.

**Table 1:** Demographic data of patients using lacosamide.

**Characteristics**	**Patients (*N* = 38)**
Age (years)	62.0 (35.5–71.25)
Gender, *n* (%)	
Male	17 (44.7%)
Female	21 (55.3%)
Reason for LCM use	
Acute seizure	6 (15.7%)
Seizure clusters	7 (18.4%)
Status epilepticus	25 (65.8%)
Patients with previous ASM use	16 (42.1%)
Previous cardiac comorbidity	
Atrial fibrillation	4 (10.5%)
First-degree AV block	1 (2.6%)
Etiology of seizures	
Antiepileptic drug withdrawal	4 (10.5%)
Autoimmune encephalitis	6 (15.7%)
Brain tumor	1 (2.6%)
Central nervous system infection	1 (2.6%)
Electrolyte imbalance	1 (2.6%)
Hyperglycemia	1 (2.6%)
Intracranial hemorrhage	6 (15.7%)
Ischemic stroke	3 (7.9%)
Multiple sclerosis	1 (2.6%)
Head injury	3 (7.9%)
Septic encephalopathy	7 (18.4%)
Dementia	1 (2.6%)
Toxin	1 (2.6%)
Unknown	2 (5.3%)

*Continuous variables were presented as median (interquartile range)*.

*Categorical variables were presented as n (%)*.

*ASM, antiseizure drugs; LCM, lacosamide*.

**Table 2:** Concomitant use of other medications at the initiation of lacosamide.

	**Medication**	***N* (%)**
No concomitant with other ASM use		3 (7.9%)
ASM	Levetiracetam	30 (78.9%)
	Valproic acid	25 (65.8%)
	Perampanel	6 (15.8%)
	Phenytoin	3 (7.9%)
	Topiramate	3 (7.9%)
	Lamotrigine	2 (5.3%)
	Carbamazepine	1 (2.6%)
	Phenobarbital	1 (2.6%)
	Pregabalin	1 (2.6%)
	Zonisamide	1 (2.6%)
Anti-arrhythmia drugs	Bisoprolol	2 (5.3%)
	Diltiazem	2 (5.3%)
	Digoxin	1 (2.6%)
	Propranolol	1 (2.6%)
	Amiodarone +diltiazem	1 (2.6%)
Anti-hypertension drugs	Amlodipine	4 (10.5%)
	Felodipine	1 (2.6%)
	Candesartan	2 (5.3%)
	Telmisartan	1 (2.6%)
	Losartan	1 (2.6%)
	Valsartan+Hydrochlorothiazide	1 (2.6%)

*Categorical variables were presented as N (%)*.

*ASM, antiseizure medications*.

The usage of LCM was presented in Table 3. LCM was used as a first-line agent for seizure in six (15.7%) patients, second-line in 14 (36.8%), third-line in 16 (42.1%), and fifth-line in two (5.3%). LCM resulted in seizure termination in 17 (44.7%) patients. The loading dose of LCM ranged from 100–400 mg and most of our patients received 400 mg as a loading dose. For those with renal function impairment, the loading dose for LCM ranged from 200–400 mg in the eight patients with moderate impairment, 200 mg for the one with severe impairment, and 200 mg for the one with ERSD with hemodialysis. Only nine patients had side effect after loading of the LCM, including vomiting (*n* = 4), drowsiness (*n* = 4), blurred vision (*n* = 1), headache (*n* = 1), skin rash (*n* = 1), and aggravated seizure (*n* = 1).

**Table 3:** Usage of lacosamide and side effects.

**Usage of LCM**	***N* = 38**
Position of LCM	
LCM as 1^st^ line ASM	6 (15.7%)
LCM as 2^nd^ line ASM	14 (36.8%)
LCM as 3^rd^ line ASM	16 (42.1%)
LCM as 4^th^ line ASM	0 (0.0%)
LCM as 5^th^ line ASM	2 (5.3%)
LCM was the last ASM to stop the seizure	
LCM was 1^st^ line ASM to stop the seizure	3 (7.7%)
LCM was 2^nd^ line ASM to stop the seizure	7 (18.4%)
LCM was 3^rd^ line ASM to stop the seizure	6 (15.7%)
LCM was 4^th^ line ASM to stop the seizure	0 (0.0%)
LCM was 5^th^ line ASM to stop the seizure	1 (2.6%)
Loading dosing of LCM	
400mg	26 (68.4%)
300mg	1 (2.6%)
200mg	10 (26.3%)
100 mg	1 (2.6%)
Side effects	*N* = 9
Vomiting	4 (10.5%)
Drowsiness	4 (10.5%)
Blurred vision	1 (2.6%)
Dizziness	1 (2.6%)
Headache	1 (2.6%)
Skin rash	1 (2.6%)
Aggravated seizure	1 (2.6%)
Cardiac side effects	
First degree AV block	1 (3.0%)
atrial premature complex	1 (3.0%)
Hypotension	1 (2.6%)

*Categorical variables were presented as n (%)*.

*ASM, antiseizure drugs; LCM, lacosamide*.

ECG was performed in 33 out of 38 patients, not all patients received ECG due to the retrospective design of this study. The five patients without ECG data were still included for the analysis of the change in blood pressure. As for ECG change after LCM loading, one (3.0%) patient had new-onset first-degree AV block and one (3.0%) had atrial premature complex in the followed ECG, both of them received 400 mg loading of LCM. The patient with new-onset first-degree AV block was treated for cluster seizure and received LCM as the third ASM after valproic acid and levetiracetam. The patient with a new atrial premature complex was treated for acute seizure and received LCM as the second ASM after levetiracetam. Neither vital signs changes nor requirement for further cardiac management was noted in these two patients with ECG change after LCM loading. Of the five patients with underlying cardiac disease, only one patient with underlying atrial fibrillation developed a new atrial premature complex. Bradycardia was not observed in our cohort. One (2.6%) patient developed hypotension (systolic blood pressure= 85 mmHg) after loading LCM, but the condition resolved spontaneously without the use of inotropic agents. Three patients stopped the LCM due to vomiting, drowsiness, and aggravated seizure. One patient stopped LCM due to septic shock that occurred 12 days after loading of LCM.

Twenty-five patients received LCM for SE treatment and nine of them were stopped by LCM (36%). Among the 25 SE patients, there were 14 patients with convulsive SE, nine focal SE, and two non-convulsive SE. Among them, one out of three patients responded to LCM as first-line therapy, three out of eight patients as second-line therapy, four out of 12 patients as third-line therapy, and one out of two patients as fifth-line therapy. Seven patients used LCM for seizure clusters, and six patients (85.7%) had seizure termination, including one out of one patient who responded to LCM as first-line therapy, three out of three patients as second-line therapy, and two out of three patients as the third line. For acute seizures, six patients received LCM, and two (33.3%) had seizures terminated by LCM, including one out of two patients who responded to LCM as first-line therapy and one out of three patients as second-line therapy.

The quantitative changes in ECG parameters and blood pressure before and after LCM loading were presented in Table 4. A total of 33 patients had completed ECG studies with four patients having atrial fibrillation which prevents the measurement of PR intervals. All 38 patients had blood pressure monitoring, which was the routine in our NICU setting. For the ECG parameters, including QRS complex, corrected QT intervals, and heart rate, there were no significant changes pre- and post-LCM loading. On the contrary, the PR interval was mild but statistically significantly increased (the average difference was 6 msecs) after loading of LCM (*p* = 0.025). A statistically significantly mild decrease in diastolic blood pressure (*P* = 0.016) and mean arterial pressure (*p* = 0.02) were also noted after LCM loading but no significant change was observed for the systolic blood pressure (*p* = 0.087). None of the PR interval changes or BP changes required medical intervention or leads to the termination of LCM therapy.

**Table 4:** The change of cardiac parameters after lacosamide use.

**Parameters**	**Baseline**	**After loading LCM**	***p*** **^#^**
Electrocardiography			
PR interval (ms)	146.0 (134.0–166.0)	152.0 (135.0–177.0)	0.025
QRS complex (ms)	82.0 (78.0–88.0)	84.0 (77.5–88.5)	0.757
Corrected QT interval (ms)	449.0 (433.0–464.8)	447.0 (428.0–455.5)	0.513
Heart rate (bpm)	92.5 (83.8–109.5)	91.0 (80.0–106.3)	0.112
Blood pressure			
Systolic BP (mmHg)	135.5 (119.0–153.0)	132.0 (111.0–143.0)	0.087
Diastolic BP (mmHg)	78.0 (66.0–86.5)	68.0 (60.0–81.0)	0.016
Mean arterial pressure (mmHg)	96.0 (87.0–108.3)	92.7 (76.7–102.0)	0.020

*The electrocardiography was obtained from 33 out of 38 patients that had received the exam*.

*Electrocardiography performed in 33 patients while 4 of them had atrial fibrillation that PR interval was unable to evaluate. Therefore, the data of PR interval was derived from 29 patients with available PR interval and other parameter of electrocardiography was calculated from all 33 patients*.

*Continuous variables were presented as median (interquartile range)*.

*BP, blood pressure; CI, confidence interval; LCM, lacosamide*.

^#^*Wilcoxon Signed-Rank Test was conducted to compare the differences of the parameters*.

In our studied population, 16 patients were aged > 65 years, eight were in the early-elderly range (65–74 years), and eight were in the late-elderly range (>75 years) (23). In the late-elderly group, four were more than 80 years. Among the elderly patients, two (12.5%) had underlying cardiac manifestation with one atrial fibrillation in the early-elderly group and the other with atrial fibrillation in the late-elderly group. The two patients that had new-onset first-degree AV block and atrial premature complex after LCM loading were the early-elderly patients. No other serious cardiac side effects were observed in the elderly patients.

## Discussion

In this study, we evaluated the cardiac safety concern in real-world epilepsy patients requiring intravenous LCM to control their seizures. Near half of this cohort responded to LCM therapy and there was no life-threatening cardiac adverse effect. One patient developed a new-onset first-degree AV block (PR>200 ms) and one hypotension (85/57 mmHg) after LCM injection, but both were asymptomatic and no subsequent management was required. Although the alterations of PR interval as well as diastolic and mean blood pressure changes were statistically significant, the degrees of changes were relatively mild in clinical practice (6 msec, 10 mmHg, and 3.3 mmHg, respectively). Considering the risk and benefit for patients with SE or seizure clusters, who may suffer from long-term cognitive impairment and/or systematic complications (15), intravenous LCM appears to be a relatively safe option in terms of cardiac concerns.

In addition to the brain, the VGSC plays an important role in the heart, especially in maintaining cardiac rhythms. Although there are different sodium channels expressed predominantly in cardiomyocytes (Nav1.5) (24) vs. neurons (Nav1.1, Nav1.2, and Nav1.6) (2), the effect of sodium channel blocking ASMs on cardiac sodium channels remains largely unclear. Theoretically, disruption of cardiac sodium channels may result in arrhythmia or conduction block, which should be monitored. This issue has been raised recently by the FDA based on *in vitro* study. LCM is a new ASM that enhances the slow inactivation of the VGSC, which is different from the old ASMs in that they enhanced the fast inactivation. Whether slow inactivation of the cardiac sodium channel is more at risk for cardiac arrhythmia than fast inactivation is completely unknown.

Our study was in line with two previous studies comparing rapid intravenous push vs. intravenous piggyback administration of LCM where the incidence of cardiac, neurological, and infusion-site adverse events was insignificant between the two methods (17, 18). These studies suggest that LCM could be given as fast as 80 mg/min, which could save the preparation time and cost. Similarly, hypotension was reported in 2.8–10% of the two studies, our study observed one patient (2.6%) with asymptomatic hypotension requiring no intervention. Bradycardia was reported in 0.7–2.6% of patients but none was seen in our cohort. Cardiac conduction defects such as second- and third-degree AV block, atrial flutter/atrial fibrillation, and bundle branch block have been associated with LCM before (25–31). In our study, mild PR prolongation and new-onset first-degree AV block after LCM were observed, but neither of them caused clinical symptoms nor needed discontinuation of LCM or required pacemaker implantation. This is in accordance with the findings from previous clinical trials of intravenous LCM (32–34). The recent study performed by Kim et al. (6) used a faster loading rate of giving 400 mg of LCM in 10 to 20 min. They showed a relatively higher rate of cardiac adverse events (32.9%) in 85 patients, especially first-degree AV block (22.4%) and prolonged mean PR interval (from 169.3 msec to 184.5 msec). The possible reason for less adverse effect in our study might be longer infusion time compared with the Kim et al. study. A side effect profile comparison of our study and Kim et al. was presented in Table 5.

**Table 5:** Comparison with previous study by Kim et al.

	**Present study (2022)**	**Kim et al. (6)**
Number of cases	38	85
Cardiac adverse effect		
First-degree AV block	*n* = 1 (3.0%)	*n* = 19 (22.4%)
Hypotension	*n* = 1 (2.6%)	*n* = 7 (8.2%)
Atrial premature complex	*n* = 1 (3.0%)	*n* = 0
Atrial fibrillation	*n* = 0	*n* = 2 (2.4%)
Bradycardia	*n* = 0	*n* = 2 (2.4%)
Atrial flutter	*n* = 0	*n* = 1 (1.2%)

Elderly patients are more susceptible to cardiac side effects of LCM (6, 34), however, we did not observe more cardiac complications in our elderly patients. Although the number of elderly patients in our cohort was small to give a solid conclusion, we consider LCM relatively safe to use in elderly patients when weighted against the devastating consequence of seizures. Most patients with renal or hepatic impairment might not receive LCM in our NICU. Since these conditions may alter the metabolism of LCM (35) and theoretically produce unexpected side effects, the treating physician may be reluctant to use LCM in these patients. In our cohort, 10 patients had renal impairment and one with elevated liver function test. From this small number of patients with renal or hepatic comorbidity, it was difficult to conclude the safety profile in this patient group.

Several limitations existed in our study. As a retrospective study, the patient selection may be biased, such that patients with a higher risk of developing cardiac side effects might not receive LCM from the treating physician. Extreme elderly patients, patients with moderate liver or renal dysfunction, and patients with cardiac disease other than arrhythmia were not included in our study which prevents further evaluation of LCM safety in these patient groups. The presence of side effects, changes in ECG parameters, or changes in blood pressure may not be fully elucidated in our small sample size. Although small in number, our cohort represented a patient group with a more critical clinical condition that required intensive care. Last but not least, patients may be unconscious in NICU and the reported side effects may be underestimated in our study. Therefore this study focused on identifying objective parameters, including ECG data and blood pressure measurements, for the evaluation of cardiac safety.

Our data revealed LCM was relatively safe for critically ill patients. Besides, we used 30 min for LCM infusion, and the result suggested this infusion rate was relatively safe in terms of cardiac side effects without compromising its effectiveness in controlling seizures. However, for some emergent conditions such as SE, a more rapid infusion rate might be needed. Further study may focus on if a shorter infusion time of LCM could be safe.

## Conclusion

Taken together, our study demonstrated that intravenous LCM does have some effects on the ECG parameters and blood pressure but is not a great concern clinically. Caution should be paid to patients with known conduction block, structural heart disease, impaired renal or hepatic function, or elderly patients. The current study is limited by the small sample size but it still provides a glimpse of the important safety issue of using LCM in NICU. Further large monitoring studies are warranted. Despite the mild concern of cardiac adverse effects in patients without obvious conduction block, the benefit of seizure control should be weighted when treating patients with SE or seizure clusters, such as hyperthermia, pulmonary edema, cardiac arrhythmias, or cardiovascular collapse.

## Data Availability Statement

The datasets presented in this article are not readily available because due to the nature of this research, participants of this study did not agree for their data to be shared publicly, so supporting data is not available. Requests to access the datasets should be directed to Y-TL, yantimlu@yahoo.com.tw.

## Ethics Statement

The studies involving human participants were reviewed and approved by the Chang Gung Medical Foundation Institutional Review Board (IRB No. 202100716B0). Written informed consent for participation was not required for this study in accordance with the national legislation and the institutional requirements.

## Author Contributions

Y-TL contributed to clinical data analysis and draft of the manuscript. C-HL, C-JH, and C-WH had contributions to clinical data acquisition and analysis. M-HT had substantial contributions to the conception and design of the study, data analysis, critical revision, and final approval of the manuscript. All authors have read and approved the final manuscript.

## Funding

The study was supported by the Chang Gung Medical Foundation (CMRPG8K0632), the Ministry of Science and Technology, Taiwan (MOST110-2314-B-182A-076-MY3), and the National Health Research Institute, Taiwan (NHRI-EX110-11022NI) to M-HT.

## Conflict of Interest

The authors declare that the research was conducted in the absence of any commercial or financial relationships that could be construed as a potential conflict of interest.

## Publisher's Note

All claims expressed in this article are solely those of the authors and do not necessarily represent those of their affiliated organizations, or those of the publisher, the editors and the reviewers. Any product that may be evaluated in this article, or claim that may be made by its manufacturer, is not guaranteed or endorsed by the publisher.
